# Implementing remote patient monitoring in lung transplant care: A real-world evaluation

**DOI:** 10.1016/j.jhlto.2026.100563

**Published:** 2026-04-16

**Authors:** Ali El Mokahal, Kelly M. Pennington, Alexander S. Niven, Desiree Neumann, McKenzie Heilskov, Darren Christopherson, Cassie C. Kennedy

**Affiliations:** a Division of Pulmonary and Critical Care Medicine, Mayo Clinic, Rochester, MN; b William J. von Liebig Center for Transplantation and Regenerative Medicine, Mayo Clinic, Rochester, MN; c Center for Digital Health, Mayo Clinic, Rochester, MN

**Keywords:** Lung transplantation, Remote patient monitoring, Telemedicine, Home spirometry, Post-transplant care, Adherence

## Abstract

**Background:**

Lung transplant recipients require close post-transplant surveillance, which imposes substantial travel and resource burdens. Remote patient monitoring (RPM), which uses home-based transmission of patient symptoms and physiologic data, has demonstrated benefit in other populations; however, data in lung transplantation remains limited.

**Methods:**

We conducted a single-center observational study of lung transplant recipients enrolled in a 12-month RPM program at Mayo Clinic Rochester. Patients used home devices to transmit symptoms, vital signs, and spirometry. Prespecified abnormal values generated alerts that were reviewed and escalated as appropriate. Primary outcomes included adherence, early discontinuation, and the impact of escalations on management.

**Results:**

One hundred and sixteen patients were enrolled. By December 2025, 47 (40.5%) completed the program, 15 (12.9%) discontinued participation, and 54 (46.6%) remained monitored. The median distance from the transplant center was 234 miles (IQR 99-385). Four hundred and seventy escalation alerts were generated, most commonly due to declines in FEV₁ (58.3%). Most escalations (72.8%) were managed with continued monitoring, while 27.2% prompted changes in care, including expedited clinic visits, diagnostic testing, medication adjustments, or emergency evaluation. Among 64 admissions with transmitted data, 31 (48.4%) were preceded by an RPM escalation in the prior 7 days.

**Conclusions:**

RPM is feasible and acceptable for post-transplant surveillance and can identify meaningful changes in health status and may be particularly useful with geographically dispersed patients. Nearly half of admissions with transmitted data were preceded by an escalation, but a substantial proportion were not, highlighting opportunities to optimize adherence and alerting thresholds.

## Background

Lung transplantation is a standard therapy for patients with advanced lung disease.^1^ Following transplantation, recipients require intensive longitudinal surveillance to detect allograft dysfunction, infection, medication toxicity, and other complications related to immunosuppression. This follow-up is complex and resource intensive. Although many patients temporarily relocate to transplant centers in the early postoperative period to facilitate frequent clinic visits, imaging, rehabilitation, and laboratory testing, ongoing subacute and long-term surveillance imposes a substantial travel, time, and financial burden on patients and caregivers. As visit intervals lengthen, clinically meaningful changes may go undetected between scheduled encounters.

Remote patient monitoring (RPM) leverages home-based devices to collect and transmit symptoms and physiologic data to clinical teams,^2^ enabling more frequent assessment in addition to traditional in-person visits. In non-solid organ transplant populations, RPM has been associated with earlier recognition of clinical deterioration,^3^ reductions in hospital utilization,^4^ and improvements in adherence and symptom detection.^5^ By extending care into the home environment, telemonitoring may also help mitigate geographic and access-related disparities,^6^ particularly for patients who live at a distance from transplant centers.^7^

Despite these potential benefits, evidence supporting RPM in lung transplant recipients remains limited. Lung transplant patients represent a uniquely high-risk population, characterized by fragile allograft function, frequent infectious and rejection-related complications, and a need for timely detection of subtle physiological changes. Prior small studies in lung transplantation suggest that remote monitoring may reduce hospital admissions and readmission-related costs,^8^ but real-world data describing program implementation, patient engagement, and clinical impact are sparse.

In this study, we describe the acceptability, feasibility, and early clinical impact of a multi-parameter RPM initiative implemented at a high-volume lung transplant center, with a unique emphasis on alert burden and downstream effects on clinical management.

## Materials and methods

This study was reviewed by the Mayo Clinic Institutional Review Board and determined to be exempt from further review under IRB protocol 25-011225. The authors agree and confirm that the study adheres to the principles of the Declaration of Istanbul and to the principles of the ISHLT Statement on Transplant Ethics. All research activities were conducted in compliance with the Minnesota Research Authorization requirements. Only data from patients who had provided appropriate authorization for the use of their medical records for research purposes were included. This study is reported in accordance with Strengthening the Reporting of Observational Studies in Epidemiology guidelines^9^; the completed checklist is provided as a supplementary file (Supplemental Table 1).

### Study design and population

This was a single-center observational study of adult lung-transplant recipients enrolled in an RPM initiative at the Mayo Clinic Rochester Transplant Center. Our practice requires relocation to Rochester, MN, for patients and 1 caregiver for 90 days following lung transplant, or for patients with extended initial hospital length-of-stay for at least 30 days of outpatient care following hospital dismissal (whichever is longer). Patients were prospectively enrolled in the outpatient setting close to the time of dismissal from Rochester—between 2.5 and 4 months after transplantation—from August 1, 2024, through December 4, 2025. In addition, patients who had undergone transplantation more than 4 months prior to program initiation were retrospectively contacted for enrollment, beginning with patients transplanted on November 25, 2023, to transition from our prior home spirometry-only program to this RPM program. Patients were excluded if they had limited English proficiency (due to reliance on English language questionnaires and equipment), required monitoring in an inpatient skilled nursing facility, were unable to transmit data from home due to lack of supportive internet/cellular services, or if the patient had financial concerns related to insurance coverage for the RPM program. Planned enrollment was 12 months for each enrollee.

### Remote monitoring intervention

Participants received an RPM kit containing a pulse oximeter (Nonin Medical Inc., Plymouth, MN), scale (Welch Allyn, Skaneateles Falls, NY), spirometer (MIR USA, Inc., New Berlin, WI), blood pressure cuff (Welch Allyn, Skaneateles Falls, NY), and a cellular-enabled Samsung Galaxy Tablet (Suwon, South Korea) for completion of symptom questionnaires and synchronization of data with the electronic patient record. Patients received in-app training for home spirometry.

All physiological and symptom data were transmitted directly to the electronic medical record and monitored by the Center for Digital Health RPM nursing team. Prespecified thresholds for abnormal values generated electronic alerts, which were reviewed by RPM nurses. Abnormal weight was defined as ≥1.0 kg/day (2.2 lbs/day) for 2 consecutive days or 2.3 kg/week (5 lbs/week). Abnormal temperature was defined as ≥100.4 F. Abnormal systolic blood pressure was defined as BP ≥ 160 or < 90 mmHg. Abnormal oxygen saturation was defined as SpO2 < 90%. Abnormal heart rate was defined as >110 bpm at rest. Spirometry baseline was defined per ISHLT criteria.^10^ Spirometry was defined as abnormal if there was an FEV1 decrease of 10% or greater from baseline. RPM nurses utilized a pre-specified decision tree to react to abnormalities including contacting patients. Patients requiring urgent evaluation were directed to their local Emergency Department. Confirmed abnormalities were forwarded to lung transplant nurse coordinators according to pre-specified severity and duration of abnormality. A transplant RN coordinator could escalate concerns to transplant physicians when clinically indicated. Participants were enrolled in the RPM program for up to 12 months, after which a physician formally assessed program completion and, when clinically appropriate, transitioned participants to a self-monitoring phase. Patients were instructed not to utilize RPM during hospitalizations, nursing home stays, or visits to the Transplant Center. For insurance coverage purposes, the minimum required transmission of RPM data was pre-specified as 17 data elements per month.

### Data collection

Data were abstracted by the Center for Digital Health team (D.N.) and included demographics, enrollment and program completion dates, vital signs, symptom questionnaire responses, home spirometry, weights, and all RPM-triggered escalations. Discontinuations were categorized as either successful program completion as approved by the lung transplant provider team, death, or early discontinuation. For patients who discontinued early, the electronic medical record was reviewed to confirm the reason for discontinuation. Primary outcomes included adherence rates, early termination rates, and changes in clinical management prompted by RPM escalations.

### Compliance assessment

Compliance was evaluated separately for vital sign submissions, spirometry, and symptom questionnaires. Vital-sign compliance was defined as the proportion of required entries completed across the cohort, and the proportion of vital-sign submissions generating alerts was also recorded. Questionnaire compliance was defined as the proportion of completed symptom surveys.

### Escalation definitions and review

An escalation was defined as any instance in which a physiological or symptom entry triggered an alert to the transplant coordinator, nursing team, or transplant physicians. For each escalation, the patient’s chart was independently reviewed in duplicate by 2 pulmonologists (A.E. and K.M.P.).

1. **Reason for escalation:** The abnormal parameter or survey entry that triggered the alert. Reasons were categorized as FEV₁ decrease, pulmonary symptoms, extra-pulmonary symptoms, weight change, or abnormalities in vital signs. When multiple abnormalities were present, the primary reason documented by the RPM nursing staff was recorded.
2. **Expedited assessment:** Whether the escalation resulted in an expedited transplant clinic visit, an emergency department evaluation, referral to another clinical service, or hospital admission.
3. **Testing:** Any unplanned diagnostic tests—such as imaging, laboratory studies, spirometry, or invasive testing—ordered as a direct result of the escalation.
4. **Intervention:** For escalations not requiring in-person evaluation, any therapeutic intervention directly resulting from the alert, including medication changes, procedures, or other management adjustments.

Each escalation was evaluated to determine whether it influenced clinical management, which we defined as any escalation that led to expedited evaluation, unplanned diagnostic testing, or therapeutic intervention.

All non-elective hospitalizations occurring during the monitoring period were also reviewed in the medical record to determine whether an RPM notification or RPM data transmission occurred within the 7 days preceding the Emergency Department visit or direct admission. The proportion of hospitalizations preceded by an RPM notification was also calculated.

### Statistical analysis

Descriptive statistics were used to summarize participant characteristics, compliance, and escalation outcomes. Continuous variables, including age, enrollment duration, and compliance rates, were summarized using means with standard deviations for normally distributed data or medians with interquartile ranges for non-normally distributed data. Categorical variables, including sex, enrollment status, and escalation categories, were reported as counts and percentages. Statistical analyses were performed using IBM SPSS Statistics for Windows, Version 29.0 (IBM Corp., Armonk, NY) and Microsoft Excel, Version 16.0 (Microsoft Corp., Redmond, WA). To assess agreement between home and lab spirometry, home spirometry recordings performed within 7 days of lab-based spirometry testing were compared using Bland-Altman analysis.^11^ For each patient, the most recent home FEV1 measurement paired with the corresponding in-laboratory spirometry measurement was used.

## Results

Baseline characteristics are summarized in Table 1. Between August 1, 2024, and December 5, 2025, a total of 116 lung-transplant recipients were enrolled in the RPM program. The most common indication for transplantation was restrictive/interstitial lung disease, accounting for 83 patients (71.6%), followed by chronic obstructive pulmonary disease in 24 patients (20.6%), and pulmonary hypertension in 9 patients (7.8%). The majority of patients underwent bilateral lung transplantation (104 patients, 89.6%). Less common procedures included unilateral lung transplantation in 3 patients (2.6%), combined heart–bilateral lung transplantation in 7 patients (6.0%), bilateral lung and kidney transplantation in 1 patient (0.9%), and combined heart, bilateral lung, and kidney transplantation in 1 patient (0.9%).

**Table 1:** Baseline Characteristics of Lung-Transplant Recipients Enrolled in the Remote Patient Monitoring Program (August 2024-September 2025)

Characteristic	Value
*Total enrolled, n*	116
*Age, years*	
Median (IQR)	63.5 (58-67)
*Sex, n (%)*	
Male	75 (65%)
Female	41 (35%)
*Indications for transplant, n (%)*	
Restrictive/interstitial lung disease	83 (71.6%)
COPD	24 (20.6%)
Pulmonary hypertension	9 (7.8%)
*Type of Transplant, n (%)*	
Bilateral lung	104 (89.6%)
Unilateral lung	3 (2.6%)
Heart and bilateral lung	7 (6.0%)
Bilateral lung and kidney	1 (0.86%)
Heart, bilateral lung and kidney	1 (0.86%)
*Monitoring status at time of analysis (Nov 2025), n (%)*	
Active	54 (46.6%)
Completed	47 (40.5%)
Dropped out	15 (12.9%)
*Geographic location, n (%)*	
Out of state	63 (54%)
Within state	53 (46%)

**Figure 1:**
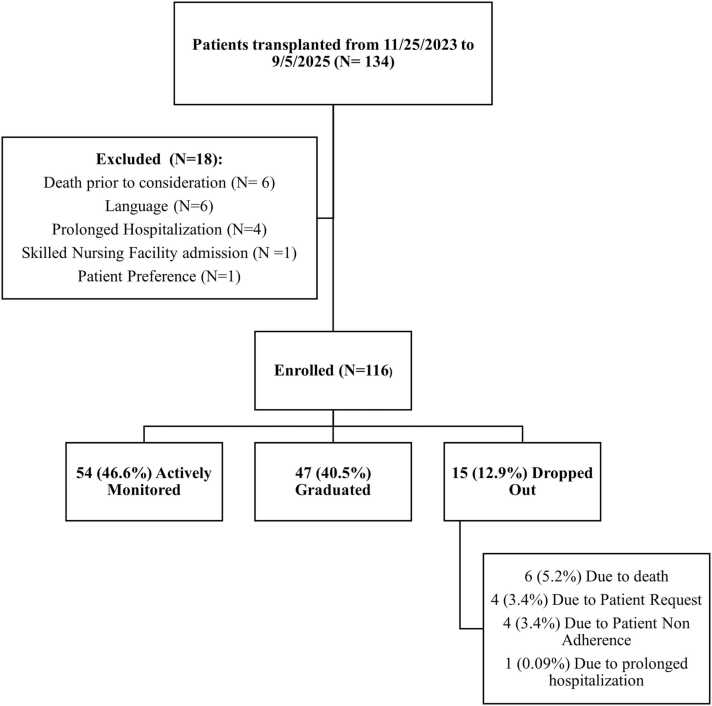
Flow diagram of participant enrollment, exclusion, program completion, and discontinuation in the remote patient monitoring program.

More than half of participants (*N* = 63, 54%) resided outside the state of Minnesota. The median distance from the transplant center was 234 miles (interquartile range (IQR) 99-385) The median age was 63.5 years (IQR 58-67 years), with 75 (65%) men and 41 (35%) women. At the time of analysis in December 2025, 54 patients (46.6%) remained actively monitored, 47 (40.5%) successfully completed the program, and 15 (12.9%) had discontinued participation. The median duration from transplantation to remote monitoring 91.5 (IQR 82.8-185.5). Figure 1 illustrates patient enrollment, exclusion, completion, and dropout throughout the study period.

Among patients who completed the RPM program, the median duration of enrollment was 351 days (IQR 216-366 days). Among the 15 patients who discontinued participation, reasons for dropout included death (*n* = 6, 5.2%), patient request (*n* = 4, 3.4%), non-adherence (*n* = 4, 3.4%), and prolonged hospitalization (*n* = 1, 0.9%).

### Engagement and compliance

Patient engagement with the RPM program was high. Completion rates for required data submissions were 62.2% for vital signs, 58.0% for spirometry submissions, and 89.8% for symptom questionnaires. Across 681 patient-months of monitoring with recorded vital-sign measurements, the median number of submissions for vital signs and spirometry per patient was 172 (IQR 118-204). Only 30 patient-months (4.4%) had fewer than 17 vital-sign entries, the minimum threshold required for insurance coverage of RPM services.

### Agreement between home and lab spirometry

**Figure 2:**
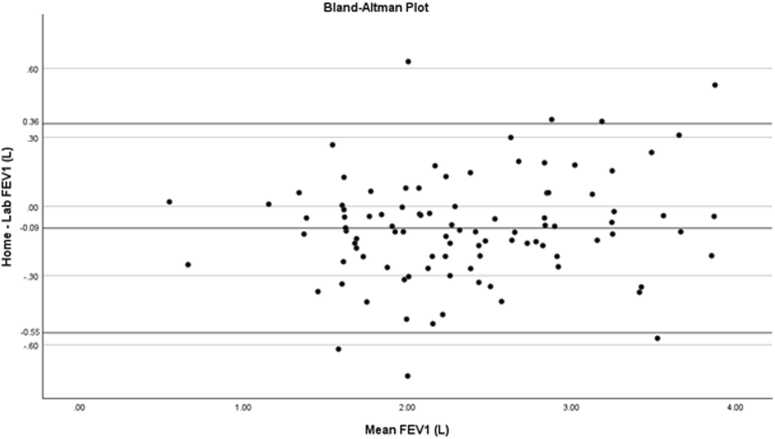
Bland-Altman plot showing the mean difference between home and laboratory measurements (middle line = −0.09) and the 95% limits of agreement (upper and lower solid lines, 0.36 and −0.55, respectively). Each point represents a paired measurement, plotted as the difference between methods against their mean.

For 96 pairs of recordings, our mean difference between lab-based spirometry and home spirometry was −0.09 L, indicating slightly lower FEV1 values measured at home. The 95% limits of agreement ranged from −0.55 to 0.36 L, reflecting moderate variability between measurements at the individual level. The Bland-Altman plot is shown in Figure 2.

### Escalations and alerts

Notifications were generated by 1.99% of vital-sign submissions and 1.91% of questionnaire responses, resulting in 497 escalations to the medical team. After excluding 27 escalations related to follow-up or program completion assessments and patients who discontinued RPM early, the median number of escalations per patient was 3 (IQR 1-7), with 80 patients (79%) experiencing at least 1 escalation.

**Figure 3:**
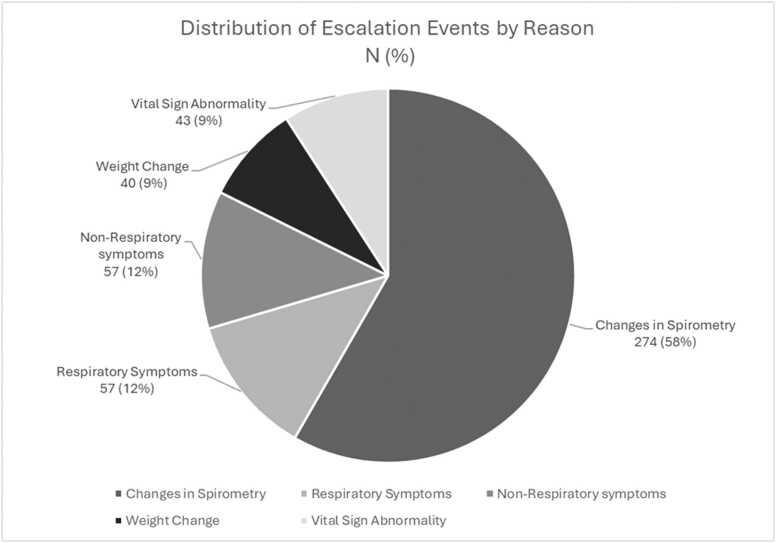
Distribution of remote patient monitoring escalation events by trigger, including declines in FEV₁, respiratory and non-respiratory symptoms, weight changes, and vital sign abnormalities. Labels are reported as *N* (%).

Declines in FEV₁ accounted for most escalations, followed by patient symptoms. Weight changes and vital sign abnormalities were less common reasons for escalation. Figure 3 shows the relative distribution of escalation events by trigger. In 16 cases (3.4%), escalation resulted in adjustment of the patient’s baseline FEV₁ value.

### Clinical impact of escalations

Management changes that occurred as a result of escalation events are detailed in Table 2. Most escalations (342 events, 72.8%) were managed with continued monitoring without changes in care, whereas 128 events (27.2%) prompted changes in clinical management. Emergency department evaluation occurred in 8 cases (1.7%) and hospital admission in 5 cases (1.1%). Among non-emergent escalations, 41 patients (8.7%) underwent expedited clinic visits, 70 (14.9%) received additional diagnostic testing, and 47 (10.0%) had therapeutic interventions.

**Table 2:** Clinical Outcomes Associated With Remote Patient Monitoring Escalation Events

Outcome of escalation events *N* = 470	Number (%)
Continued monitoring without a change in care	342 (72.8%)
Any change in care	128 (27.2%)
Testing ordered	70 (14.9%)
Therapeutic interventions ordered	47 (10.0%)
Emergency department evaluation	8 (1.7%)
Hospital admission	5 (1.1%)
Expedited clinic visits	41 (8.7%)
Baseline spirometry value adjusted	16 (3.4%)

### Hospitalizations and mortality

During the monitoring period, 73 non-elective hospital admissions occurred. In 9 admissions (12.3%), vital sign data were not submitted in the week preceding hospitalization. Among the remaining 64 admissions, 31 (48.4%) were preceded by an RPM escalation within the prior 7 days. The most common presenting reasons for non-elective hospitalization were dyspnea (23 admissions), fever (8 admissions), chest pain, diarrhea, and failure to thrive (4 admissions each), followed by vomiting (3 admissions). Less frequent presenting complaints included falls, syncope, fatigue, dysuria, abdominal pain, palpitations, and a variety of single-occurrence symptoms.

Among the 6 patients who died during the study period, 2 patients were enrolled but had not begun transmitting, and the third patient stopped transmitting due to a recent hospitalization. Of the remaining 3 patients, 2 patients had an RPM escalation preceding hospital admission, while 1 patient did not.

## Discussion

RPM has been used successfully for oncology patients in the setting of covid-19 and febrile neutropenia.^12^, ^13^ Our findings support the use of RPM as a feasible and clinically meaningful adjunct to post-lung transplant care. This initiative uniquely demonstrates that multi-parameter RPM—integrating spirometry, symptoms, and vital signs with structured nursing oversight can be sustained over a full year with high patient acceptance and meaningful clinical impact.

Enrollment and retention rates were high, and engagement was strongest for symptom questionnaires, while adherence to physiologic monitoring was more moderate. These findings are consistent with prior studies examining adherence to home monitoring and telehealth programs in transplant populations.^14^, ^15^, ^16^, ^17^, ^18^, ^19^ For example, Sengpiel et al found that 73% of patients demonstrated adherence rates greater than 80%, while 25% had adherence rates between 50% and 79%.^15^ The higher rate of adherence in that study is attributable to the fact that spirometry was the sole required measurement. In contrast, Odisho et al described a multi-parametric monitoring application but noted lower participation rates for symptom reporting and spirometry transmissions.^18^ Similarly, studies evaluating patient satisfaction have reported high levels of acceptability and perceived benefit with remote monitoring technologies.^20^, ^21^, ^22^ The relatively lower adherence to physiologic measurements observed in our cohort warrants further investigation, particularly given prior evidence linking higher adherence to home spirometry with reduced overall costs^19^ and lower rates of chronic lung allograft dysfunction.^23^ Lower adherence may in part reflect the burden associated with multi-device monitoring and the extended duration of participation (1 year). While daily measurements were encouraged, this expectation may have been challenging for some patients; nevertheless, less frequent data capture (e.g., weekly or twice-weekly measurements) remained informative and clinically useful.

Declines in FEV₁ accounted for most alerts, underscoring the continued importance of home spirometry as a cornerstone of lung transplant surveillance. Prior studies have demonstrated good correlation between home and laboratory-based spirometry,^17^, ^24^, ^25^ earlier detection of rejection,^26^, ^27^, ^28^, ^29^ opportunistic infection,^26^ and improved clinical outcomes.^23^ Subsequent investigations expanded beyond spirometry alone to include patient diaries, symptom questionnaires, and digital monitoring platforms,^14^, ^18^, ^22^, ^30^, ^31^, ^32^ as well as structured nursing triage and decision-support system.^30^

Unlike earlier home spirometry programs,^33^ this RPM initiative incorporated multi-parameter monitoring, including home spirometry, vital signs, weight trends, and patient-reported symptoms, all integrated into the electronic medical record with structured nursing review and escalation pathways. Alerts for weight changes, vital sign abnormalities, and symptoms were also common, and a meaningful proportion of these resulted in changes in management.

A unique insight from this work is that alerts generated through RPM frequently prompted clinically relevant changes in patient care and demonstrated the downstream consequences. Although most escalations were managed with continued monitoring alone, nearly one-third resulted in changes in clinical management, including expedited clinic visits, diagnostic testing, medication adjustments, or emergency evaluation. This is expected in high-risk populations, as we want the sensitivity for alerts to be high. The number of alerts needed for 1 change in management was approximately 4. These findings highlight RPM’s ability to detect physiological and symptomatic changes that may not otherwise come to clinical attention between scheduled visits, enabling earlier clinical response. While a substantial proportion of alerts did not prompt immediate action, in lung transplantation—where delayed recognition of rejection, infection, or allograft dysfunction can have rapid and catastrophic consequences—a higher alert burden may represent an acceptable tradeoff for patient safety, particularly in the early post-transplant period. Frequent in-person assessments, routine laboratory testing, and ongoing patient self-monitoring contributed to a substantial proportion of alerts not resulting in a documented management change, as the clinical issues prompting these alerts may have already been identified and addressed during scheduled encounters.

The potential for RPM to anticipate clinical deterioration is further supported by the finding that 48% of non-elective hospitalizations were preceded by an escalation within the prior 7 days. In our review of the escalations, only 5 escalations prompted direct hospitalization because of the notification. The rest may have been reflections of evolving disease that was already destined to require admission. Additionally, it is important to note that assessing whether RPM prevented hospitalization is beyond the scope of this observational study. Furthermore a considerable number of hospitalizations were not preceded by alerts, reflecting variability in patient adherence, limitations in monitoring sensitivity, or the abrupt onset of complications not readily detectable through RPM. These findings highlight the need for ongoing iterative refinement of monitoring parameters and escalation pathways as RPM programs mature.

Although formal cost-effectiveness analyses were beyond the scope of this study, cost considerations are important aspects affecting the sustanability of RPM in lung transplantation. In a retrospective analysis of home monitoring in lung transplant patients, Adam et al demonstrated that adherence was associated with substantial reductions in cost.^34^ Broader evidence from chronic disease and post-acute care populations also support these findings, a systematic review by Tan et al reported that RPM interventions are frequently associated with a decrease in health care costs, although outcomes are heterogenous.^5^ This data suggests that meaningful reductions in healthcare costs are feasible with RPM when programs are appropriately designed and effectively implemented.

Finally, this initiative demonstrates the potential for RPM to extend the reach of transplant programs beyond traditional clinical encounters. With more than half of enrolled patients residing outside our geographic region, RPM may help mitigate geographic disparities in access to high-quality post-transplant care by reducing reliance on frequent in-person assessments. By filtering large volumes of physiologic and symptom data and routing actionable findings to clinical teams, RPM may also support more efficient use of transplant program resources, allowing clinicians to focus attention on patients with evolving clinical needs.

### Limitations

This study has several limitations. As a single-center observational implementation, causal inference is limited, and findings may not be generalizable to transplant centers with different patient populations, workflows, or resources. The absence of a comparator group precludes assessment of the effect of RPM on hard clinical outcomes such as hospitalization rates, length of stay, or survival. Adherence to physiologic monitoring varied across device types, and reasons for missed entries were not systematically captured, limiting interpretation of incomplete data. In addition, not all hospitalizations or deaths were preceded by RPM alerts, reflecting both variability in adherence and the unpredictable nature of some post-transplant complications. Enrollment was limited to English-speaking patients, introducing potential selection bias and limiting applicability to linguistically diverse populations. Finally, minimum data submission requirements tied to reimbursement may have influenced patient drop-out rates and engagement.

## Conclusion

Multi-parameter RPM is a feasible and acceptable strategy for augmenting post-transplant surveillance in lung transplant recipients. By enabling frequent, structured assessment of symptoms and physiology, RPM facilitates early identification of clinically relevant changes and may help anticipate clinical deterioration. Although further work is needed to optimize alert algorithms and evaluate downstream clinical and economic outcomes, these findings support the integration of RPM into hybrid models of post-transplant care. With continued refinement, remote monitoring has the potential to enhance patient safety, mitigate geographic disparities, and strengthen the delivery of high-quality, longitudinal lung transplant care. Future studies should provide interpreter support, or alternative language options for questionnaires should be considered to mitigate bias.

## Financial support

This study received no external funding.

## CRediT authorship contribution statement

**Conceptualization:** Kelly M. Pennington, Cassie C. Kennedy. **Methodology:** Kelly M. Pennington, Ali El Mokahal, Cassie C. Kennedy. **Data Curation:** Desiree Neumann, Ali El Mokahal, Kelly M. Pennington. **Formal Analysis:** Ali El Mokahal. **Program Implementation:** McKenzie Heilskov, Darren Christopherson. **Writing – Original Draft:** Ali El Mokahal, Kelly M. Pennington. **Writing – Review & Editing:** All authors. **Supervision:** Kelly M. Pennington, Cassie C. Kennedy.

## Conflicts of Interest statement

The authors declare that they have no known competing financial interests or personal relationships that could have appeared to influence the work reported in this paper.

## Footnotes

Appendix A Supplementary data associated with this article can be found in the online version at doi:10.1016/j.jhlto.2026.100563.
