# Supplementary material for: Implementing remote patient monitoring in lung transplant care: A real-world evaluation
Source: JHLT Open. 2026 Apr 16;13:100563. doi: 10.1016/j.jhlto.2026.100563 (PMC13146546; doi:10.1016/j.jhlto.2026.100563)
Supplement: Supplementary file 1 — Supplementary material [file mmc1.docx]

**Supplementary Table 1.** STROBE Checklist for Observational Studies

| **STROBE Item** | **Recommendation** | **Manuscript Location** |
| --- | --- | --- |
| **Title and Abstract** |  |  |
| 1(a) | Indicate the study’s design with a commonly used term in the title or abstract | Abstract; Methods |
| 1(b) | Provide an informative and balanced summary of what was done and what was found | Abstract |
| **Introduction** |  |  |
| 2 | Explain the scientific background and rationale for the investigation | Introduction |
| 3 | State specific objectives, including any prespecified hypotheses | Introduction (final paragraph) |
| **Methods** |  |  |
| 4 | Present key elements of study design early in the paper | Methods – Study Design and Population |
| 5 | Describe the setting, locations, and relevant dates | Methods – Study Design and Population |
| 6(a) | Give the eligibility criteria and sources and methods of participant selection | Methods – Study Design and Population |
| 7 | Clearly define all outcomes, exposures, predictors, and potential confounders | Methods – Data Collection; Escalation Definitions |
| 8 | Describe data sources and methods of assessment | Methods – Remote Monitoring Intervention; Data Collection |
| 9 | Describe efforts to address potential sources of bias | Methods – Escalation Definitions and Review |
| 10 | Explain how the study size was arrived at | Methods – Study Design and Population |
| 11 | Explain how quantitative variables were handled | Methods – Statistical Analysis |
| 12(a) | Describe all statistical methods | Methods – Statistical Analysis |
| 12(b) | Describe methods used to examine subgroups or interactions | Not applicable |
| 12(c) | Explain how missing data were addressed | Methods – Compliance Assessment |
| 12(d) | Describe analytical methods accounting for sampling strategy | Not applicable |
| 12(e) | Describe sensitivity analyses | Not applicable |
| **Results** |  |  |
| 13(a) | Report numbers of individuals at each stage of study | Results; Figure 1 |
| 13(b) | Give reasons for non-participation at each stage | Results |
| 13(c) | Consider use of a flow diagram | Figure 1 |
| 14(a) | Give characteristics of study participants | Results; Table 1 |
| 14(b) | Indicate number of participants with missing data | Results – Engagement and Compliance |
| 15 | Report numbers of outcome events or summary measures | Results; Tables 1–2 |
| 16(a) | Give unadjusted estimates and, if applicable, adjusted estimates | Results |
| 16(b) | Report category boundaries when continuous variables were categorized | Not applicable |
| 16(c) | Translate estimates of relative risk into absolute risk if relevant | Not applicable |
| **Discussion** |  |  |
| 17 | Summarize key results with reference to study objectives | Discussion (opening paragraph) |
| 18 | Discuss limitations of the study | Limitations |
| 19 | Give a cautious overall interpretation of results | Discussion |
| 20 | Discuss the generalizability of the study results | Discussion; Limitations |
| **Other Information** |  |  |
| 21 | Give the source of funding and the role of funders | Title Page; Funding Statement |
