# Supplementary material for: Implementing remote patient monitoring in lung transplant care: A real-world evaluation
Source: JHLT Open. 2026 Apr 16;13:100563. doi: 10.1016/j.jhlto.2026.100563 (PMC13146546; doi:10.1016/j.jhlto.2026.100563)
Supplement: Supplementary file 2 — Supplementary material [file mmc2.docx]

Ali El Mokahal : [www.linkedin.com/in/ali-el-mokahal-542ba63a3](http://www.linkedin.com/in/ali-el-mokahal-542ba63a3)

Accompanying Post:

In a single-center cohort of lung transplant recipients, 12 month, multi-parameter remote patient monitoring was feasible, had high retention, and generated clinically meaningful alerts that informed care.
